# Exploring experiences of the regulatory toxicology system: system-level promoters and inhibitors of new approach methodologies

**DOI:** 10.1007/s00204-025-04168-z

**Published:** 2025-09-09

**Authors:** Angela Bearth, Birgit Kopainsky, Lowenna B. Jones, Gunn E. Vist, Trine Husøy, Camilla Svendsen, Paul Whaley, Sebastian Hoffmann, Heather M. Ames, Gisle Solstad, Denise Bloch, Aleksandra Čavoški, Weihsueh A. Chiu, Miles Davenport, Holly G. Davies, Arianna Giusti, Thomas Hartung, Seok Kwon, Olivia J. Osborne, Andrew A. Rooney, Christophe Rousselle, Jennifer B. Sass, Fred A. Wright, Gro H. Mathisen

**Affiliations:** 1 Norwegian Scientific Committee for Food and Environment, Norwegian Institute of Public Health, Oslo, Norway; 2 HF Partners, Tièchestrasse 63, 8037 Zurich, Switzerland; 3 System Dynamics Group, University of Bergen, Bergen, Norway; 4 Department of Politics and International Relations, University of Sheffield, Sheffield, UK; 5 Division for Health Services, Norwegian Institute of Public Health, Oslo, Norway; 6 Department of Food Safety, Norwegian Institute of Public Health, Oslo, Norway; 7 Department of Chemical Toxicology, Norwegian Institute of Public Health, Oslo, Norway; 8 Lancaster Environment Centre, Lancaster University, Lancaster, UK; 9 Evidence-Based Toxicology Collaboration (EBTC), Bloomberg School of Public Health, Johns Hopkins University, Baltimore, MD USA; 10 seh consulting + services, Paderborn, Germany; 11 Department of Pesticides Safety, German Federal Institute for Risk Assessment (BfR), Berlin, Germany; 12 Birmingham Law School, University of Birmingham, Birmingham, UK; 13 Department of Veterinary Physiology and Pharmacology, College of Veterinary Medicine and Biomedical Sciences, Texas A&M University, College Station, TX USA; 14 Kirby Institute, University of New South Wales, Sydney, NSW Australia; 15 Washington State Department of Health, Tumwater, WA USA; 16 Cosmetics Europe, Avenue Herrmann-Debroux 40, 1160 Auderghem, Belgium; 17 Center for Alternatives to Animal Testing (CAAT), Bloomberg School of Public Health, Johns Hopkins University, Baltimore, MD USA; 18 CAAT Europe, University of Konstanz, Constance, Germany; 19 Department of Pharmacology, Yong Loo Lin School of Medicine, National University of Singapore, Singapore, Singapore; 20 Chemical Risk Assessment Team, Science, Evidence and Research Division, Food Standards Agency, Petty France, Westminster, London, SW1H 9EX UK; 21 Division of Translational Toxicology, National Institute of Environmental Health Sciences, Research Triangle Park, NC USA; 22 ANSES/DAEI-French Agency for Food, Environmental and Occupational Health & Safety, 14 rue Pierre et Marie Curie, 94701 Maisons-Alfort Cedex, France; 23 Natural Resources Defense Council, Washington, DC USA; 24 Departments of Statistics and Biological Sciences and Bioinformatics Research Center, North Carolina State University, Raleigh, NC 2695 USA

**Keywords:** Next generation risk assessment, New approach methodologies, Chemical risk assessment, Systems thinking

## Abstract

The transition from traditional animal-based approaches and assessments to New Approach Methodologies (NAMs) marks a scientific revolution in regulatory toxicology, with the potential of enhancing human and environmental protection. However, implementing the effective use of NAMs in regulatory toxicology has proven to be challenging, and so far, efforts to facilitate this change frequently focus on singular technical, psychological or economic inhibitors. This article takes a system-thinking approach to these challenges, a holistic framework for describing interactive relationships between the components of a system of interest. In this case, the regulatory toxicology system. We do so by analysing and interpreting a very large qualitative data set of experts’ observations, collected in a 3-day interactive workshop and three follow-up online workshops with a heterogeneous sample of experts representing major actors from the global regulatory toxicology system. We identified leverage points (where a small change within a system can have a disproportionately large effect) in the six core aspects—infrastructure, processes, culture, technology, goals, and actors—in the regulatory toxicology system to facilitate the effective use of NAMs. Identified systematic leverage points include the need for a functioning incentive structure for effectively discovering, developing, validating and using NAMs within academia, regulation, and industry; and measures that prevent or mitigate unwanted effects of using NAMs that acknowledge clashes between scientific, regulatory, political and social processes. The results serve as a basis for follow-up activities that reflect on the actual effectiveness of these levers and that develop measures for the regulatory toxicology system.

## Introduction

The transition from traditional animal-based approaches and assessments to New Approach Methodologies (NAMs) marks a scientific revolution in regulatory toxicology (Cattaneo et al. 2023; Hartung and Tsatsakis 2021; National Research Council 2007; Schmeisser et al. 2023). A paradigm shift in generating, integrating, and interpreting NAMs data needed to perform hazard and risk assessments holds the promise of enhancing human and environmental protection by making chemical safety assessment higher-throughput, cost-effective and offer a way to improve the mechanistic understanding of effects on the human system (Auerbach et al. 2024; Judson 2018; Manful et al. 2023; Simon et al. 2024). Yet, the steps necessary to make the shift happen successfully have proven challenging. While existing assessments and summaries have acknowledged the difficulties associated with a paradigm shift in a functioning system (Archibald et al. 2015; Čavoški et al. 2023; Sewell et al. 2024), they frequently only offer a snapshot or very specific aspects driving or inhibiting the uptake of NAMs (Bearth et al. 2025; Schiffelers et al. 2012). Most importantly, the goal is often framed as “increasing the acceptance and use of NAMs,” leading to tensions between institutions and individuals pressing to phase-out and replace animal testing with NAMs, and those that call for patience and sufficient characterization to ensure that NAMs offer equal or better protection than the current system before NAMs are adopted for widespread use (European Chemicals Agency 2023).

We propose a new perspective on this controversial field by identifying system-level factors that facilitate or inhibit integration of academic innovation and regulatory science and policy into a well-functioning regulatory toxicology system. These system-level factors are less obvious as they span across different experiences of individuals working within the regulatory toxicology system (Meadows 2008; Monat and Gannon 2023), which was addressed in this study by qualitatively analysing the reported experiences and beliefs of a group of experts with diverse backgrounds working in or around the regulatory toxicology system. Two conceptual frameworks served as a backdrop for our study: a systems-thinking approach (Meadows 2008) and the socio-technical systems (STS) model (Davis et al. 2014; Hendrick 2006). These two conceptual approaches are introduced subsequently.

### A system thinking approach to the regulatory toxicology system

System thinking is a holistic framework for describing interactive relationships between the components of a system (Meadows 2008). In this case, the system of our interest is the regulatory toxicology. In general, prior approaches to understanding NAM uptake tend to focus on specific components such as

- validation (e.g., reliability, relevance) or filling gaps in the underlying science (methodological factors) (Browne et al. 2024; Holzer et al. 2023; Osborne et al. 2024),
- the psychology of the people working in the regulatory toxicology system (social factors) (Bearth et al. 2024, 2025; Mondou et al. 2020; Pain et al. 2020; Zaunbrecher et al. 2017),
- and/or economic factors (Meigs et al. 2018).

While providing relevant information on methodology, psychology, or economics, these studies only partly capture the complexity and multitude of interested parties operating in the regulatory toxicology system. A systems-thinking approach broadens the horizon of inquiry, capturing system-level factors. In this case, system-level factors are aspects of the regulatory toxicology system in which NAMs are developed and NAM data is collected, interpreted and acted on. These system-level factors are potentially changeable through well-placed systemic interventions (Senge 1990). However, such system-level factors can be difficult to assess, as actors in the system only experience parts of the system, not necessarily the system in its entirety. For example, the perspective of an academic researcher will reflect experiences in the discovery and design of NAMs, use them to generate data, publish them, and be on the applicant side (and possibly evaluation side) of funding programs. In contrast, an employee of a regulatory agency may have experience with what NAM data is suitable for a particular application, what it is like to develop health advisories or enforce laws based on NAM data and address potential challenges from regulated parties, sometimes in the form of litigation. Thus, system factors are hidden within the lived experiences of the people working within a system (e.g., events witnessed, experiences made, beliefs of how the system works or should work) and can only be accessed by combining input from groups working in different areas of the regulatory toxicology system and exploring differences and similarities (Eker and Zimmermann 2016; Rajah and Kopainsky 2025).

### Utilising the socio-technical systems model to classify system-level factors

The socio-technical systems (STS) model (Davis et al. 2014; Hendrick 2006) offers a framework for classifying and differentiating system-level factors. The framework is built on the foundation of general systems and open systems theory (Emery 1959; von Bertalanffy 1950) and evolved since its inception (Trist 1981). The STS model, at its core, considers that a system can only be understood and improved if both technical aspects (i.e., *infrastructure*, *technology*, *processes*) and social aspects (i.e., *culture*, *goals*, *actors*) are taken into account as inter-dependent parts of a complex system (Davis et al. 2014; Mumford 2000). The term *actors* here are not just individuals but also groups of people (such as academic, regulators, journalism/media) as well as organizations/institutions. The STS model considers that within a system, there are actors with capabilities, who work toward goals, follow processes, use technology, operate within a physical infrastructure and share certain cultural assumptions and norms.

We use the STS model to structure the nuanced intermediate objectives for the effective use of NAMs in regulatory toxicology. Thus, the goal of *interventions* (a purposeful, planned action to change the system) in the regulatory toxicology system should be to facilitate regulatory *infrastructures*, *processes*, *culture* and *technology* that increase the effectiveness of the use of NAMs, according to the *goals* of the *actors* in and outside the system (Chartres et al. 2022; Mathisen et al. 2024). Put simply, we want to promote better use of NAMs that “should be used”, because they offer actionable evidence (i.e., a scientific basis for health-protective actions), improvements to the status quo, or other benefits. We also want to prioritise those NAMs over those that “should not be used”, because they do not provide actionable evidence, are not reproducible, are inefficient, or do not have a ready fit-for-purpose application. This perspective could potentially move the regulatory toxicology system away (faster) from the undesirable state of ineffective use of NAMs towards the desired state of effective use of NAMs.

## Overarching project and manuscript research goals

The interdisciplinary CHANGE project (Collaboration to Harmonise the Assessment of Next Generation Evidence) adopts a system-based perspective to developing system-level interventions for effective NAM adoption in regulatory toxicology (Mathisen et al. 2024). CHANGE comprises a three-phase methodological approach that focuses on the views of the global regulatory toxicology system as expressed by the people working in it. The overall approach is summarised in Mathisen et al. (2024).

In the first phase of the CHANGE project, the “Explore Phase,” we arranged a 3-day in-person workshop, and a series of follow-up online workshops for invited people with different roles within the regulatory toxicology system to share anecdotes about their experiences of working in this system and the role that NAMs might potentially play in it (Jones et al. n.d.). These anecdotes offer an inroad into participants’ lived experiences of working in the regulatory toxicology system through the recounting of events (i.e., issues catching our attention) and patterns (i.e., repeated events shaped by the underlying structure). There is an inherent subjectivity in these anecdotes, due to the underlying differences in personal beliefs and values, which means they may reflect less how the system is supposed to work and more how the system works from the perspectives of the people in it. Collecting and comparing multiple anecdotes about the same aspect of the regulatory toxicology system allows access to the less observable structures, meaning the way in which system components interact and thus, form the infrastructure, processes, technology, culture, goals and not least, actors of and within the system. This article summarises the themes that emerged from the “Explore Phase,” focusing on collecting people’s observations about the regulatory toxicology system and joining these disparate events and patterns together to form an initial theory of the underlying structures, i.e., how the regulatory toxicology system works, considering the current paradigm shift.

The CHANGE project will then follow this “Explore Phase” with two subsequent phases: the Reflect Phase and the Design Phase. Both these phases also feature interactive workshops with people working in the regulatory toxicology system (Mathisen et al. 2024). In the “Reflect Phase,” the initial theory of the underlying structures summarized in this manuscript will be presented and refined. In the “Design phase,” system-level interventions will be suggested to promote the effective use of NAMs.

## Methodological approach

**Fig. 1:**
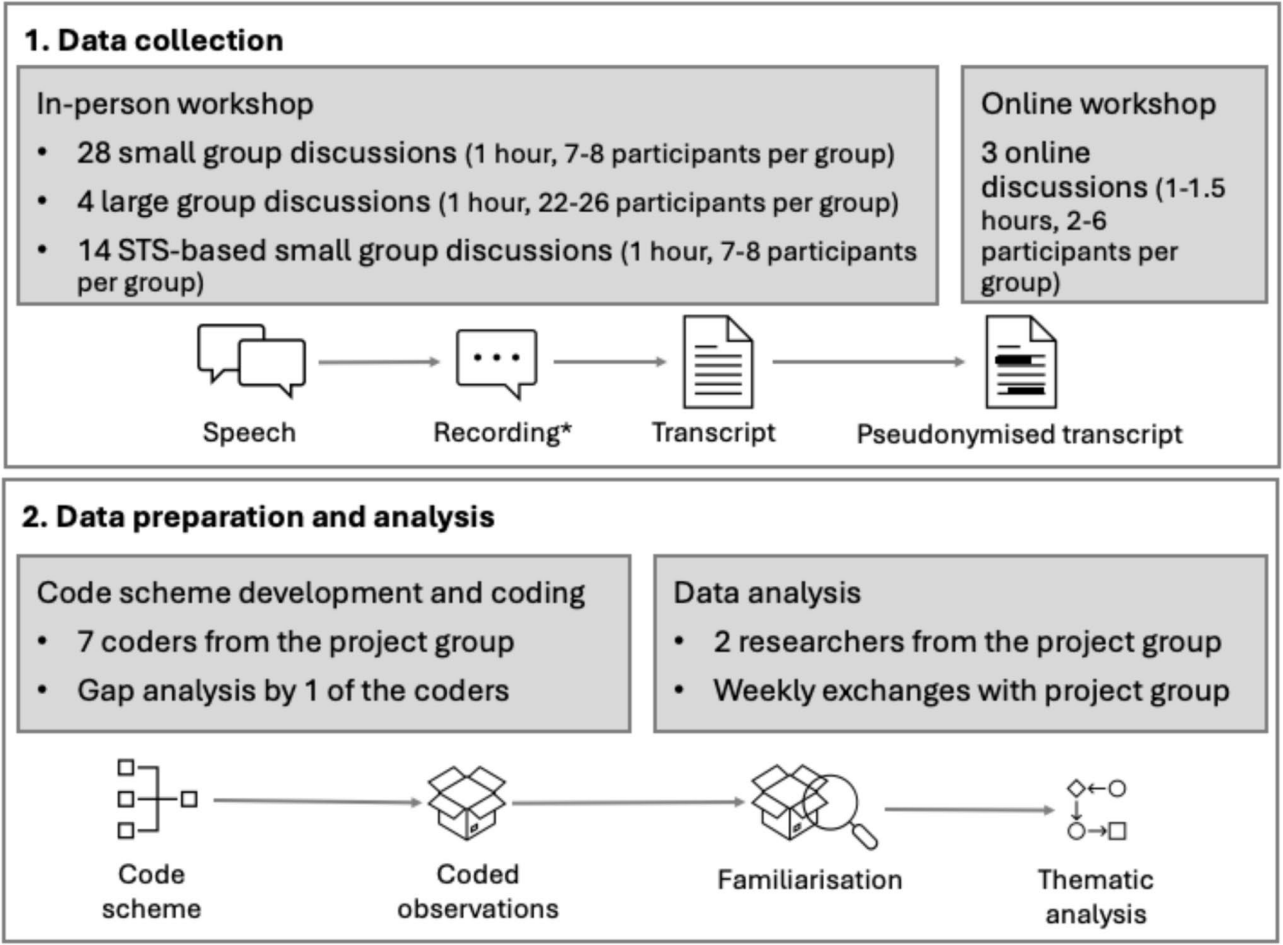
Overview of methodological approach. *STS* socio-technical systems. *To protect the participants’ privacy and to encourage open sharing in the workshop the recording was maintained for the limited time necessary to produce the transcript and then destroyed

Figure 1 presents an overview of the methodological approach. We present our methods based on the overall design of the workshops (Section “Design of the workshop”); the data collection in the workshop itself, including session structure and recording of data (Section “Data collection”); preparation of the data from the workshop for analysis, including transcription and pseudonymisation (Section “Data preparation”), and analysis of the data itself, including qualitative analysis methods and coding strategy (Section “Data analysis”).

### Design of the workshop

Data were collected in a three-day in-person workshop in Oslo, Norway in June 2024, and in three online workshops in October 2024. Care was taken to recruit a heterogeneous group of workshop participants that covered a broad spectrum of activities related to NAMs development, conduct, and potential consideration in the regulatory context. This group had diverse professional roles (i.e., risk managers, risk assessors, researchers), institutional backgrounds (i.e., industry, national and international authority or agency, academia, consultancy, non-governmental organisation, non-profit organisation), discipline (i.e., human health and environment) and legal framework (e.g., plant protection products, biocides, industrial chemicals, cosmetics, medicinal and veterinary products, food additives). The workshop participants were from 16 countries in Europe, two countries in Asia, and one country each in North America, South America, Australia. All workshop participants received a detailed information pack prior to participating (Mathisen and Solstad 2025). Participants signed informed consent forms and agreed to be recorded during the workshop. A Data Protection Impact Assessment was performed and approved by the Norwegian Institute of Public Health. Ethics approval was waived as no sensitive information was being collected and no more than minimal risk was posed to participants given the agreement to destroy recordings once a written transcript was generated and pseudonymised.

### Data collection

The in-person workshop featured several types of collaborative sessions—small group discussion, “fishbowl” discussion, and STS-based small group discussion—to collect as many anecdotes about the regulatory toxicology system as possible.

At the start of the first 2 days, four 60-min small *group discussions* with seven groups ran in parallel served to collect an initial set of anecdotes. This resulted in a total of 28 group discussion sessions. The group sessions were preceded by four input presentations from workshop participants on different topics that aimed at sparking discussion. The topics of the input presentations were (a) regulatory siloes, (b) protection vs. prediction, (c) academic vs. regulatory data and (d) standard operating procedures. Group and moderator assignment was randomised using an online tool for team generation (Random Lists 2018) to vary group composition.

Large group discussions, so-called “fishbowl discussions,” were held at the end of the first 2 days. This resulted in a total of 4 fishbowl discussion sessions. Each fishbowl discussion featured an inner circle with 4–6 chairs (fishbowl) surrounded by the audience. Participants could leave the fishbowl whenever they wanted and could enter the fishbowl when a chair became free, but were only allowed to participate in the discussion when they were in the fishbowl. One moderator and 22–26 workshop participants were randomly assigned to one of the groups for each of the sessions to vary group composition using the same online tool as for small group discussion (Random Lists 2018). Each day, two separate 60-min *fishbowl discussions* were run in parallel to keep group sizes manageable. These sessions served to expand on existing anecdotes and spark new ones. At the start of the fishbowl discussion, 4–5 participants were invited by the moderator to join the inner circle based on the anecdotes they had told in the small group discussions.

On the third day, the last type of interactive sessions served to refine existing anecdotes. For this, the participants were asked to digitally rate the anecdotes in an open session according to which ones they wanted to discuss more, found surprising, and/or thought of as controversial. The prioritised anecdotes (criteria for prioritisation: one anecdote received votes for discussion more and surprising, while the remaining anecdotes that were selected received votes for one of the three criteria: most interesting, surprising, and controversial) were then selected for discussion in two consecutive 60-min *STS-based small group discussions* with 7 groups running in parallel. This resulted in a total of 14 group discussion sessions. In these STS discussions, the participants were asked to analyse the chosen anecdote by identifying the elements from the STS model in “node maps”. Node maps are a visualisation representation using nodes, lines between nodes, and text (in six colours indicating six core aspects of the socio technical systems (STS) model. See “Utilising the socio-technical systems model to classify system-level factors” section) to show the interconnections of aspects/elements within a system. The participants were asked to prepare these node maps for their assigned anecdote.

The follow-up online workshops served to include participants that were not able to attend the in-person workshop and thus, to diversify the backgrounds of the participants. Three 60–90-min online workshops, applying the small group discussion methodology from the in-person workshop were run with one moderator and 2–6 participants per workshops.

### Data preparation

All anecdotes were recorded ad hoc on sheets of paper by the moderators of the session and complemented in written format by the workshop participants who introduced the anecdote. All collaborative sessions were recorded. The recordings in the in-person workshops were made by professional sound technicians with Microsoft Teams recordings as back-ups. The recording in the online workshops were recorded using Microsoft Teams (Microsoft 2024) and audio files were separated from the video-recordings by using a converter (Aiseesoft Studio 2024). The transcripts were transcribed using Whisper (OpenAI 2024) and manually pseudonymised by removing identifiers, such as names of participants, institutions and chemicals.

Thus, the data basis for this article was the 48 transcripts of the in-person workshop sessions and the online workshops (about 43 h of transcribed recordings). Observations about the regulatory toxicology system provided in the anecdotes were coded according to a coding system developed collaboratively, founded in the STS model. After familiarisation with the transcripts, six coders (AB, GM, PW, GV, TH, CS) each generated an individual code scheme based on the transcripts of two fishbowl discussions. An additional coder (LJ) developed a code scheme based on the node maps generated in the STS-based small group discussion sessions on the third day of the workshop. These diverse code schemes were discussed among all coders (AB, GM, PW, GV, CS, TH, LJ) and one coder (AB) developed the final code scheme.

The code scheme (see in Table 3 in the Appendix) features the six core elements of the STS model, including descriptions and anchor examples (cf. Appendix). Two additional codes, “solid gold” and “unsure” allowed the coders to add a code to observations that they thought were noteworthy or that did not fit into the code system to alleviate the familiarisation step in the data analysis due to the extensiveness of the available qualitative data. Seven coders then coded the transcripts using ATLAS.ti (Lumiverso/ATLAS.ti Scientific Software Development GmbH 2024). The group discussions were only coded once by one coder, while the fishbowl discussion sessions were coded by at least two coders independently. This resulted in 1310 coded observations. The coders engaged in weekly exchanges to address difficulties emerging during the coding and align the approach to the coding. One coder compared the fishbowl discussions that were double-coded and consolidated divergences. Additionally, one coder went through a randomly selected set of five transcripts for a gap analysis, i.e., to gauge the observations that might have been missed by the initial coders. This gap analysis revealed that from these five randomly selected transcripts, 15 of 190 observations were missed (8%). Additional checks at the end of the data analysis showed that all 15 observations were already reflected in other coded observations; the same anecdotes or anecdotes about the same topic were told in multiple sessions.

### Data analysis

The data analysis was led by two researchers, one with a social science and risk research background (AB) and the other with a system thinking background (BK), through weekly exchanges with the interdisciplinary project group. The method of data analysis was based on thematic analysis, relying on comparing observations that were assigned the same code, uncovering similarities and developing hypotheses about reasons for differences in observations (Mayring 1991). After familiarisation with the material per code, themes were identified and reviewed in discussions with the project group. The analysis featured the inspection of overlaps among codes and sub-codes, i.e., observations that were coded onto two or more (sub)codes. The results and interpretations of the results were substantiated by presenting selected observations, which have been lightly edited for clarity or to ensure the protection of the participants’ identity by removing speech mannerisms or highly identifiable examples. Such edits are indicated in the quotes by square brackets.

## Results

### Overview of observations and linkages between codes

**Fig. 2:**
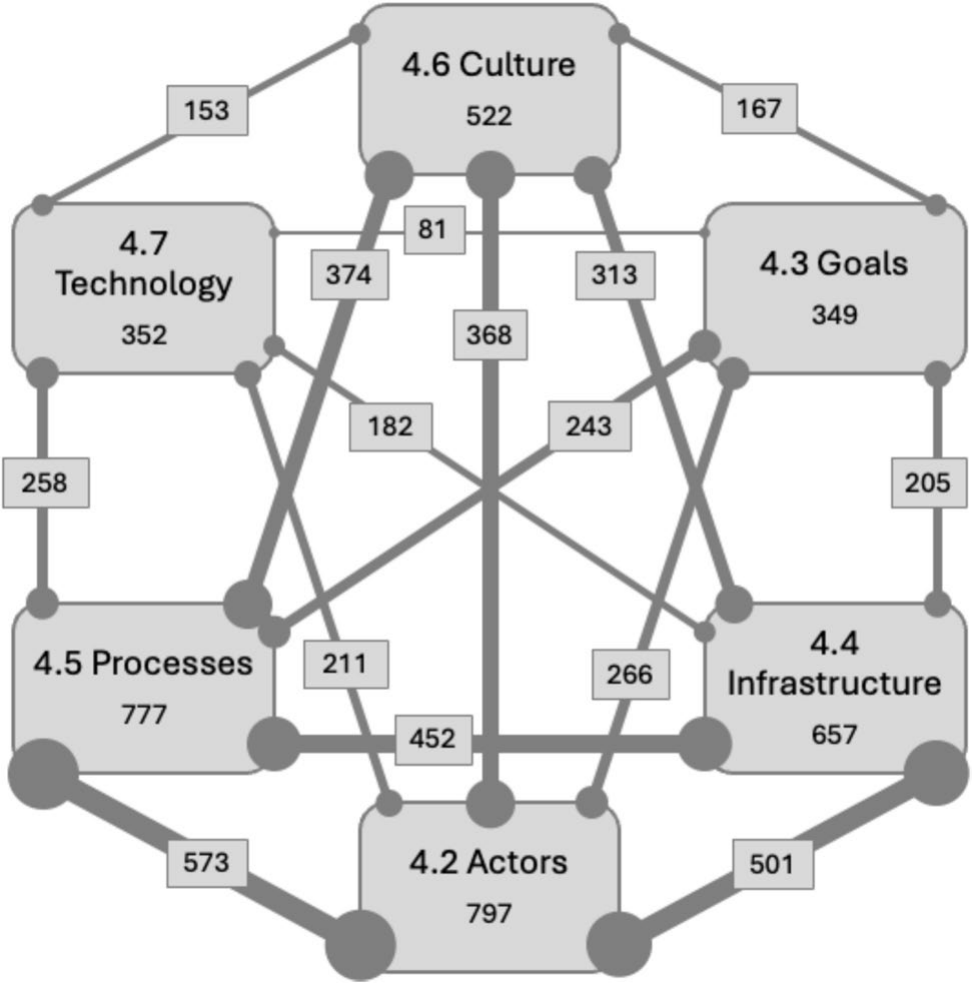
Observations pertain to each aspect of the system (or per code) and linkages among aspects/codes (width of lines and radius of circles indicate the number of linkages)

The richness of the collected data allowed for some initial quantitative analysis showing the number of observations per code and linkages between codes (Fig. 2). Among the six aspects of STS, *processes*, *actors*, and *infrastructure* had the most observations, followed by *culture*, *goals*, and *technology*. Most prevalent were linkages between actors, processes and infrastructure. However, the frequencies should be interpreted with care. Observations that are made more frequently by different or the same participants^1^ could suggest a more universal topic throughout the regulatory toxicology system, or simply a more familiar issue among participants. Observations made less frequently may suggest less important issues, or issues that are more complex or well-hidden across the experiences of different people working in the regulatory toxicology system. The subsequent sub-sections cover each of the six aspects from Fig. 2, while also acknowledging the linkages between elements of the STS model.

### Observations about actors

Numerous different **actors** were mentioned in the observations of the participants. It is important to note that while the anecdotes frequently featured experiences made with individuals, the category “actors” also represents groups of people, institutions, or organisations, not just individuals. Table 1 provides an overview of the number of observations that featured one or more actors from a particular group, with academia and the scientific community and regulators being the most frequent.

**Table 1:** Number of observations about different actors

Actor	Includes	Frequency
Academia and the scientific community	Scientific researchers of all levels, funding agencies, and scientific publishers	370
Regulators	Risk assessors and managers, regulators, and regulatory agencies	353
Industry	Manufacturers, and producers	247
Government	Regulatory agencies, politicians, and policy makers	204
Public	Exposed communities, and lay-people	83
Legal system	Lawmakers, lawyers, and judges	47
Non-governmental organisations (NGOs)	NGOs, non-profit organisations (NPOs), and public interest groups	42
Media or journalists	Media, journalists, and other public commentators	20

While some of these actors were discussed as an integral part of the regulatory toxicology system (e.g., regulators, industry), others were discussed more about the pressures that they apply to the system, frequently based on their respective goals (i.e., mission of academic and scientific community, government, NGOs, media or journalists, public, legal system).

### Observations about goals

A **goal** is the broad purpose of an actor, such as the reason for the existence of an institution and the driving force of a profession. Different actors might follow congruent or conflicting goals, and the same actor might simultaneously follow conflicting goals. Many observations suggest that conflicting goals or even just the expectations of conflicting goals can hamper dialogue and collaboration within the regulatory toxicology system. The goals of each actor play a large role in determining the (expected) purpose of NAM. We further want to stress that the definition of goals, as done by our workshop participants, does not necessarily represent universal truths, but rather individual definitions of one’s own goals or speculative goals of other actors in the system. Thus, individuals might be somewhat unreliable narrators when discussing their own or other actors’ goals. One of the key messages from observations related to goals is that NAMs data are used by different actors with varying goals for a multitude of purposes.

#### Goals of regulators

The goal of regulators was frequently described as *protecting human health and the environment* or *following and implementing statutory requirements*. This overarching goal may or may not conflict with other sub-goals inherent to the institution (e.g., *assessing risks to human health and the environment, minimizing risk of legal or scientific challenges)* or the goals of other actors involved in the infrastructure and processes associated with regulatory toxicology (e.g., *promoting the use of NAMs, meeting regulatory requirements)*.

#### Goals of academia

A prevalent goal of academia was described as *conducting research*, and was frequently linked to higher purposes, such as scientific progress and gaining an understanding of the world around us. Accordingly, NAMs are discovered and developed by academics as a means of innovation or as a tool to investigate a research question of interest, with or without regulatory interest.

*“I think there's a lot of good science out there that not only isn't regulatory science, but it really shouldn't have to be because it should be cutting edge. It should be something new and undefined. It should be the Star Trek mission of science, […] I do want to go where no one's gone before. That is the mission of science, is to explore new places and not to use existing protocols and three doses, 10, 100, and 1,000 times apart […]”*

*“ […] scientists were specialists […] and it was not about a method but it was to work on why and how a disease arises and then, to answer this question, you start to work on models but it's a complete different set of mind […] our research is built in the entire world […] and toxicology is a really small part. We are here between us, and we imagine that you know [toxicology] is the alpha and the omega, but many labs are there just here to understand about a disease.”*

*“I think that's the beauty of the academic research that you have these degrees of freedom to just find out what's there in front of you and dig into something that looks interesting.”*

#### Goals of industry

The goals of industry were described as *bringing products and services to market and making profit*, thus, the purpose of the use of NAMs is to test new chemicals (or those already in use) effectively and efficiently, obtain regulatory approval, or fulfil a market need or consumer request (i.e., animal-free safety testing)*.*

*“I've been, a few years ago, trying to get a [new product] approved […] We didn't want to do any animal testing on this ingredient for various reasons, ethics, but also from a claims-point of view, you know, vegan classification requires that no animal testing is done on the ingredient. So, we developed a strategy, which was based on not doing any animal testing.”*

*“And as some of our industrial stakeholders claim, we would very much like to use NAMS, but if there are no regulatory requirements, we will not use money on it.”*

*“We were working with a third-party ingredient supplier […] and that ingredient supplier decides to do the animal test because they felt it was quicker and higher chance of success. They were short of money, and they needed the ingredient quickly, approved quickly so they could sell it to other customers.”*

#### Incentives for all actors

The overarching goals determine the most relevant **incentives** for institutions and individuals. These incentives are embedded in the infrastructure, processes, and culture of the regulatory toxicology system, and drive actors’ views of and decisions regarding NAMs. Thus, incentives to develop, validate, accept, and use NAMs (or the lack thereof) were a prevalent theme addressed by the participants. According to the participants, incentive structures may explain issues with the effective use of NAMs. This is particularly true, as academia, industry and regulation cooperate, interact and rely on each other within the regulatory toxicology systems. For academics, it was observed that incentive structures prioritise innovative and result-focused publications or a set number of publications (e.g., for PhD students to finish their doctorate), both of which motivate splitting data. Academic researchers typically lack incentives for long-term effort that are needed for an assay to be accepted for fulfilling regulatory requirements.

*“… you get incentivized to do the newest, shiniest thing […] the newest sequencing that could be done […] you're incentivized to do that […] and that incentive means that it's letting a thousand flowers bloom, but nobody settles down to [...] ‘This is the thing we're going to study and do this for the next 10 years so that we can develop a consistent database.’”*

The development and validation of an NAM is a substantial investment, as was particularly highlighted by participants working in industry. These participants highlighted the importance of financial incentives, either through cost savings, profitable innovations or an image improvement in the public eye.

*“I get more funding for development, not for validation, so I have been working with industry, and they want to develop new methods, they want to use these new methods, but they only have a limited grant for development. So, after you develop, they show for the society that they are good, so the consumers can see that this is a better product because they are investing [in] science.”*

The events and patterns described by the participants suggest the absence of incentive structures for the use of NAMs or NAM-generated data for academics, industry and regulation, even if they offer a relevant and valid response to the regulatory question at hand. Based on some observations made by participants with heterogeneous backgrounds, one could argue that the status quo (i.e., the use of existing, well-known or animal-based methodologies) is strongly incentivised, as the participants described a steep price attached to breaking new ground in terms of methods used (e.g., resources, possible social or legal consequences of deviating from well-established practices).

*“And regulators, on the other hand, are driven by objective science because they don't have the same incentive to constantly be running after funding or fame or whatever it is that, you know, academia is driven by.”*

*“The personal experience is [...], you get a lot of […] you get resistance. You get resistance because it's sort of, it's different from what we're used to.”*

#### NAM use contexts and goals

Another issue that was raised was the different use contexts. The participants diverge in their views of whether NAMs alone could detect hazards associated with a substance, as well as whether NAMs would ever be useful in determining no risk given a certain exposure context. The observations made about these different use contexts were largely determined by the imagined use context of NAMs: Using NAMs for screening purposes or when using NAMs for making safety decisions. These use contexts have very different preconditions, evidence requirements, and implications for actors with different goals, among them concerns over false positives or negatives and negotiations regarding different levels of certainty by different actors. The co-existence, and in some cases clashes, of these expected use contexts can hamper the effective use of NAMs due to pressures applied to the system from different actors in light of the inconsistency of interpretations of NAM data. Similarly, the desired protection level linked to a particular chemical can be seen as a type of goal when it comes to the effective use of NAMs, which has been linked by many participants to the external validity of NAMs data (e.g., the extent to which the results obtained from NAMs can be generalised to real-world human or environmental health outcomes beyond the specific test conditions). For instance, the desired protection levels might be higher for chemicals approved for a particular use than for an unavoidable contaminant).

*“NAMs are interesting because they're allowed to open signals of alerts [...] But what we see is that for the moment when you have these signals, regulators do not yet dare taking regulatory measures [...] because they keep questioning the human relevance.”*

*“You always have risk assessment … and then you have risk management, look at this number, look at the risk and the benefit, and then we make the judgment. Sometimes risk/benefit, for example in food, there's no benefit, like you never […] take a risk, it's always a risk. That's the difference between like a drug […] when you take into account a risk/benefit, then obviously the level playing field and the whole game has changed, because that becomes, instead of a consumer, it becomes a patient, which it is, right? But with food, for example, it's never, it's always about risk.”*

### Observations about infrastructure

Frequently, the observations highlighted that the regulatory toxicology **infrastructure** is mostly designed for animal testing, while certain structural factors can contribute to more openness towards NAMs.

*“But we just want to replace some of the data with data that we generate with alternatives. And I think that's actually an issue because then everybody is working towards human models because we want to know what's safe for humans, but we want to plug that sort of in a patch that over a framework that is built for animal data.”*

Subsequently, the observations about infrastructure are presented in five sub-themes: dialogue, legal structures, funding, awareness, knowledge and skills, and supply chain.

#### Dialogue

Structures that support the **dialogue** and collaboration among different actors were seen as vital for the effective use of NAMs, but were also seen critically in light of potential conflicts of interest. As an example, collaborations between industry and regulatory partners in developing a NAM were described as effective due to the early and joint coordination of needs, while simultaneously not being incentivised, supported or even seen as problematic. For instance, it was criticised that industry might have an undue influence on the regulator’s decision by co-developing the rules for favourable decisions about their dossiers if collaborating too closely with regulatory partners.

*“I've seen examples from the working groups of other countries where there is more collaboration with funding between […] industrial partners[,] regulatory agencies and academics. I think that could work much more broadly but there's just a resistance, almost like there's firewalls between all of those sectors, at least in [Country] and maybe they're intentional or they're just historical.”*

*“I find that there's a lot of incentives to not have the regulatory agencies work directly with, say, the inventors of the technology, and I find that really odd. And then you'll have people who are trying to adapt what you know without really the experience to do it, and then that slows it down in their compartment, too. It's like, why not just continue to work together?”*

The infrastructure for these dialogues and collaborations must be facilitated well, as there were anecdotes that hinted at the inherent shortcomings of regulator-driven innovation. Moreover, NAM developers and regulatory risk assessors might be challenged by the inherent interdisciplinarity of developing and using a NAM, or might be faced with a chicken-and-egg-problem, as outlined in this dialogue between two participants:

*“What actions do you take?”*

*“You talk to an individual. You need to interact with the regulatory bodies and then you look for funding from the regulatory perspectives. There's like a funding mechanism available for developing this shared understanding […] then you spend the money on developing the NAMs they want.”*

*“The regulators specifically fund the NAMs that they want?”*

*“Yes.”*

*“So how do the regulators decide what NAMs they want?”*

*“Exactly.”*

#### Legal structures

According to the participants’ observations, **legal structures** can promote but also hinder the effective use of NAMs, although many participants highlighted that few legal frameworks actually preclude the use of NAMs for regulatory decision making (i.e., for regulatory acceptance or flagging potential human health or environmental risks). Because NAMs are framed as possibilities (rather than requirements), these legal considerations connect with the observation of the absence of incentives for changing the status quo, as outlined previously. Another prevalent theme discussed here is the meeting (or clashing) of legal, regulatory and scientific infrastructure. Thus, scientific and regulatory uncertainty is paired with the experience of regulators that evidence gained from NAMs does not necessarily hold up in court. This dilemma becomes particularly evident in the discussions surrounding legal and regulatory processes, detailed in the next section.

*“I think that researchers usually claim that we need to change the regulation. I think that all the regulations, […] include statements promoting the use of non-animal methods. So, we have possibilities. In most of the cases, it's the guidance that is based on animal methods, but the regulations are different.”*

*“[Institution] says no more [chemical in a specific product], which I think they might have said it anyway, and the [Chemical] industry takes them to court. And they've made their decision on cells in a petri dish, modelled with some model that nobody understands, with a lot of assumptions that aren't really listed anywhere, with a code that's only three-quarters public, because it was pulled from [another context]. And it was done by a contractor who does it for money.”*

#### Funding

**Funding** infrastructure was discussed primarily regarding the validation of NAMs. Validation for NAMs entails understanding the reliability (measure of intra- and inter-laboratory reproducibility) and relevance (usefulness of a NAM for a particular purpose). Observations at the workshop described that funding periods are too short for validation or that funders value innovation over validation. This lack of funding is exacerbated by the absence of infrastructure and processes (e.g., scientific outlets, funding instruments), culture (e.g., acknowledgment, appreciation) and goals (e.g., incentives) for academics to seek funding for validation or to validate the methodologies they developed or are using.

*“[…] academics are quite interested in validation and they would really benefit from investment and funding in validation and one thing that has changed especially in [Country], there is a great emphasis of evaluating academic work through what they call impact, so what is the impact that an academic makes on a regulatory environment, so I think at least in [Country] that is something to get engaged because this is what they can see if you're an academic you can publish papers you can get promoted, this is what sets you off.”*

*“Most of the projects are finishing this year and most of them haven't really brought anything to final validation, simply because five years is not enough time to bring something through from development to optimization to validation and test guideline development.“*

#### Awareness, knowledge and skills

Infrastructure that promotes **familiarity with, knowledge about and skills to use NAMs** within the system was seen to promote the effective use of NAMs. The participants expected that the potentially increased specialisation required for the use of NAMs hampers the effective use of NAMs. Targeted education was seen by many participants as a way to overcome this challenge, while many also cautioned over an overreliance on educational measures, while ignoring other challenges (e.g., resource restraints). Another aspect that was discussed, particularly in areas with low availability of data or high uncertainty, is the weight placed on expert judgment. For example, observations described that experienced risk assessors have gut feelings about the validity of a method or of a particular result, a skill that develops over years of practice and allows for efficient and pragmatic judgment and decision making. Only a few risk assessors were described to be sufficiently familiar with more recent or innovative NAMs or are exposed frequently enough to NAMs data to develop these skills in a similar fashion as for animal data or more established NAMs. In several observations, it became apparent that some participants expect this transition to happen without intervention and that the shift to the efficient use of NAMs would occur automatically through a focus on NAMs in academic curriculums or other training venues.

*“Many risk assessors would probably feel like their gut feeling is well-based in their expertise. Because they knew so much about it, they felt at ease to trust this gut feeling. Whereas with the NAMs, that might take a while, and then you would be too worried that your gut feeling is biased, perhaps.”*

*“The senior, the bosses there, they are trained in one system where, you can say, the animal testing was good standards. And now there is a transmission going from [this] to NAMs, and that requires a lot of training at the universities. And before they are raising up in the ranks, so to say, it's like a big ship, and you have to train it, and it goes very, very slowly.”*

While academics produce large amounts of NAMs data and are frequently involved in the development of new methodologies, the observations of the participants showed that not all academics are aware of the particularities of the regulatory toxicology system (or the legal infrastructure and processes attached to it). Participants suggest that supporting academics to develop the skills needed to translate their research into regulatory terminology and meet the necessary regulatory requirements can enable their contribution to the regulatory toxicology system, and thus, promote the effective use of NAMs.

*“We like to say that when we want to develop something, we go to the regulators because we like to understand what they need. Because we are developing something for the regulatory authorities and such. So, if you don't understand what they need, it's no point.”*

*“There's academic scientists come into this space, and they have this scrutiny and criticism, and it gets misinterpreted, but they bring up valid points, and they're trying to help the process, but it totally derails the process. Stakeholders when they hear the discussion that this method might not do 100% what people think it should, it's completely disapproval from academic world and I don't think academic scientists quite understand the impact of some of their statements when it's brought into the public space like that and so there might be a need to maybe educate academic scientists on this.”*

*“From academia data, we have the most problem is terminology because if you go through different reports, you're not sure if they're really reporting the same thing because it's not really reported well.”*

#### Supply chain

Although frequently overlooked, infrastructures of the **supply chain** can hamper the effective use of NAMs through a variety of gaps, e.g., lack of availability of cell materials or test guidelines, certified laboratories or data storage facilities. A more in-depth exploration of this issue might be of interest, as it was only mentioned superficially during the workshops.

*“Sometimes the research funding is for the innovation and when you come with a test for validation, you need engineers, technicians, a laboratory able to perform that according to quality assurance system, etc. […] Maybe we have to build a better system of laboratories. It's very difficult. Most laboratories are also not made for the more complex models. And they don't know how to handle. And then, you know, they cannot even grow the normal culture. And then you evaluate not the method, but how good is the laboratory in itself. And that is, it's a disaster. And it's one of the limits of the system as well. Because we have not a lot of laboratories able to perform. But that's the reality.”*

*“You're going to go there and say, can you move my data to new places? ‘Yes, you give me the money. I do that for you.’ So, where does the money come from? From the academia setting, that's been very difficult. And to do in the government, a little bit better. Once the money was [handed] down to us, so we will be able to maintain some of the critical data. Interesting to figure out how much cost there is in aggregate and storing data. It's quite a lot of money.”*

*“We've had examples where a test guideline was supposed to be freely available […] and then once it got adopted, the test method developer thought ‘Oh I can make lots of money. I want to start selling this. I don't want it to be freely available anymore’ […] but at the same time, when a company is investing so much into the development how are they going to generate an income back if they don't charge licensing fees, if they don't.”*

### Observations about processes

Regarding **processes**, a particular finding from the analyses of the participants’ observations is that scientific, economic, political and juridical processes frequently clash and thus, hinder the effective use of NAMs. For example, the “first mover hesitancy” has been described as the hesitancy to base a regulatory assessment on a new or not well-established method. This hesitancy links **scientific processes** (e.g., approaches to the uncertainty associated with NAM data) with **legal processes** (e.g., lawsuits, legal consequences) and **social processes** (e.g., risk aversion of the public or within institutions towards chemical risks, risk aversion towards innovation in the regulatory toxicology system, increased scepticism towards NAMs, worrying over personal consequences or backlashes within and outside of the organisation). Many observations hinted that guidance, guidelines, or official documents putting a stamp of approval on a specific NAM offer a way to relieve these dilemmas by offering clarity within an uncertain field and prescribing a particular test or method for a specific purpose. However, multiple observations suggested that this has the unwanted effects, hindering the application of potentially useful NAMs which are currently not featured in any guidance documents (e.g., through the enforcement of the status quo of using animal data or misunderstandings of guidance as legislation).

*“[…] I use that example to reflect upon the fact, that challenging the substance of a decision, challenging the risk management decision relied upon on the evidence, is very difficult to do. It's very difficult to say that your exercise of judgment is erroneous. But what is much easier to do is to say that there are procedural flaws in the data, that your data is incomplete or insubstantial.”*

*“Law is very unresponsive to change because there is a lot of inertia, you don't want to change for various reasons, it's very difficult. And as you say, it's very difficult to set new objectives.”*

Some participants described **scientific and political processes** running in parallel, where sometimes political arguments (e.g., real-life implications of banning a chemical or of setting a particular threshold value) are hidden behind flawed scientific arguments (e.g., rejecting evidence based on uncertainty in the available data). These observations highlight the need for NAM developers and users to be aware of complex interactions within the system and the need to acquire skills that go beyond the technical aspects of NAMs (i.e., skills and infrastructures and processes for developing these skills in light of the absence of an incentive structure).

*“A lot of times it's political decisions disguised as scientific arguments. So, you know, one can argue that the discussions at [institution] are very scientific and they should be. But I keep saying that acceptance of a method is not just related to validation or scientific endeavours. It's very much related also to the political situation, the needs of countries, and they might not always coincide.”*

Many participants observed that they felt an **inconsistency in regulatory processes**, which on the one hand demand a move towards NAMs due to expectations about potential benefits (e.g., testing large amounts of chemicals, testing mixtures), while simultaneously requesting animal tests (e.g., request for an oral study, despite only dermal contact to workers).

*“I mean you cannot run animal testing to make all the potential different exposures of humans or the environment to chemicals. So doing in vitro testing is really a great opportunity but when we present results of scientific literature on [NAM data] the reflex we have from national regulators is saying ‘Oh yes but we don't know what […] the human relevance [is].’”*

*“We saw even though the […] strategy to minimize the request for animal studies was already in place, [the institution] republished the guidance on [chemical], and they were asking for even more animal studies. So, this is the asynchrony of the general vision and then the reality of what is requested for applicants, I believe.”*

*“There are cases where you have a [study focused on specific exposure route], […] and the [institution] rejects [this study] and asks you for a [study focused on another specific exposure route]. That is still happening today in 2024 at the same year when […] we want the roadmap to completely get away from animal testing. And so, these are two directions which are really going in different directions. And at the moment for me, it's a double talk and this should really come to the front.”*

An important point that became apparent from the joint discussions was the **unwanted effects of using NAMs**, either previously observed or expected by the participants. Several examples of real and expected negative effects (e.g., triggering more animal studies or flagging large numbers of substances in use as a risk due to false positives when applying NAMs) are outlined in the quotes below. A few themes emerge from the discussions. First, the effective use of NAMs implies that the unwanted effects of using NAMs are avoided or that mechanisms are in place that amend them. Second, expected negative effects (i.e., beliefs and assumptions that the use of NAMs might have detrimental effects in the system, for their goals, or in light of the protection of human health and the environment) limit the openness of the regulatory toxicology system towards potentially useful NAMs. All real and expected unwanted effects were linked in the observations to the higher (perceived) uncertainty linked to NAMs, the lack of decision frameworks that deal with false positives or negatives, or the difficulty of demonstrating the absence of an effect using NAMs.

*“But our initial proposal was to increase [… the] requirements with a lot of NAM data, which could be collected and support the assessment of those low tonnage substances, and which are typically data poor, but to include much more data. The problem of what we saw was that with the discussions going forward with our colleagues from [institution] […] and others, was that those data would then actually start triggering more in vivo studies. So, the introduction of NAMs and non-animal data in the […] would actually have the opposite effect of what we wanted to see in the first place, which would be actually triggering more animal studies.”*

*“If we would have to rely purely on the in vitro assay, we would have struck out a lot more, a lot more of the compounds. So yeah, they're definitely more conservative.”*

*“If something is negative, the regulator would usually say of such an in vitro test. ‘Well, let's show and demonstrate that also in an animal test, please.’”*

*“Let's say a simple in vitro test and something has a positive result, the positive result is something that an industry would always follow up with. ‘Let's do the animal test to confirm whether or not we can believe it.’ And that's why a lot of tests are designed to screen. They are designed to have a lot of false positives. You can definitely be sure to identify the effect. But the result is that if you would take that as a fixed answer, a lot of very helpful chemicals will be not on the market. “*

*“What are we going to do with all of these compounds that are flagged? What we really need is decision frameworks to help us understand how we can use this information, right? Is one assay enough to drive to the next level? Or is it an assay that has a certain level of potency? Is it a pattern of effects? And then what would that follow up look like? What we're seeing […] right now is [NAMs are] flagging things like [chemical] and [chemical] and [chemical]. What should be negatives in at least one assay system? It'd be great to think about what that decision framework should look like so that we don't end up moving all of these to in vivo […] studies, because I don't think that's the right answer for how to really get a handle on [… the] potential [of a particular endpoint].”*

### Observations about culture

Many observations were made about the **culture** within regulatory toxicology. Culture in this context refers to the shared values, beliefs, norms, and practices among social systems, such as groups, institutions or societies. These observations can roughly be divided into events describing the **beliefs and assumptions** that were attributed to **habits or behaviours** of people and institutions of the system.

*“What I've observed of the industry focus on NAMs is: The specific things that they need, like read across to another chemical to get it approved or decreasing uncertainty factors. Those aren't necessarily, incorrect, it is just applying resources to a very, very specific question. That's not necessarily like a public health question, or it’s a more economic question.”.*

Particularly, **trust among different cultures** within the system (or lack thereof) was described as an essential component of a well-working regulatory toxicology system. This notion was not necessarily openly discussed, but was latent in the observations of the participants. In the observations, it became further apparent that the level of trust was dependent on the frequency and quality of interactions (dialogue, collaboration), as interactions allowed the participants to observe the culture first-hand. However, interactions among participants also highlighted that in the absence of first-hand experiences, trust was built more on expectations, people’s own beliefs and assumptions about the goals and incentive structures of others. Accordingly, a lack of trust was sometimes linked to a lack of transparency, such as expectations that the industry would omit NAM data that does not fit into the overall safety assessment or that academics would publish data, despite the lack of reproducibility. In other cases, the lack of trust was more related to the absence of personal interactions and relationships across groups, which would likely be trust-promoting.

*“They were afraid that the method would be misused by industry and replacing other methods they were used to. And the project got blocked.”*

*“It's a little bit complicated when you discuss with researchers, because if you discuss with the people that kind of push animal testing, then you have one way of hearing the arguments on how trustworthy these studies are, and that they will be better than the NAMs, for example. And then when you hear from researchers doing research on NAMs, and it's a little bit sensitive as well, because it's a bit personal, because you want to go away from animal testing, and then you have the arguments that you cannot trust the animal studies, … and then as a regulator, it's a bit complicated to know what to trust in a way, because you can do your own research, but this is also very clever researchers that have good arguments on each their side, and this I feel is a bit difficult with the discussions around NAMs, because you want to use the data in the best way possible”*

*“Another barrier with academia I also think is about trust. Because there is not always trust in data coming from academia. Because it's not reproducible sometimes.”*

*“So, industry often don't trust the regulators to have enough experience to understand what they're submitting, for example.”*

### Observations about technology

Few observations pertained to specific **methodologies** and even more infrequently, **tools** used within the regulatory toxicology system. Frequently, observations pitted **hazard- vs. risk-based approaches** against each other and linked primarily the former to a so-called “tick-box approach” that might preclude the effective use of NAMs in regulatory toxicology.

*“One of the key problems which leads to this tickboxing is this hazard-focused approach where you say, I can only, I'm only satisfied with my animal data if I had seen toxicity in my mother animals. If I haven't seen toxicity in your mother animals, this molecule was not tested high enough, there was not exposure enough. Even if the exposure of the animal study is a thousand times above what the consumer would be exposed, we need to see this. And that comes from this hazard assessment idea that only if I dose to a study where I see my animals even suffering, I'm sure that I did the worst case. And that is what leads to this tickboxing that studies are scrutinized not for whether they tell us whether it's safe for humans, they tell us whether we did what we believe is needed for what we call hazard assessment. And I think that is one reason which drives up the test concentration, which drives up the study numbers, which drives up the repeat studies and which brings us to this tickboxing approach.”*

Another topic that was discussed controversially was the potential for NAMs to produce more **human-or population-relevant data**. The participants criticised the extrapolation from animal data to humans and the (default) uncertainty factors traditionally associated with this transfer (i.e., factor of 100). However, not all participants agreed that NAMs would be more predictive or protective than whole animal testing. Participants also highlighted the challenge of combining multiple separate assays into a test battery. With the increasing number of assays in a test battery, the number of false positives is likely to increase.

*“We have used uncertainty factors traditionally just to extrapolate from humans, from rodents to humans, and the same uncertainty factor or the similar uncertainty factor just being chemical agnostic. Now we have the opportunity to really dig into [the] specific […] chemical entities to really put the levels of uncertainty based on the specificity of the chemical and not always the 100th uncertainty factor for interspecies or interspecies extrapolation. So that it was a concept that is so old, and I always wonder how come that such a science that is so specific for the human health is still so in the past with the uncertainty factors. And now there is an opportunity to really go beyond the uncertainty factors, be more human centric. That is also something that is absolutely powerful of next generation risk assessment because we will focus using NAMs and to get information that are specific for the humans and not anymore relying in these animal tests that not always are as informative of the human situation as we believe.”*

*“We all know that there is no solution for [endpoint] because the [testing] is really expensive. It's not very predictive. Very few people do it. And it's funny to see that in the absence of a solution, having something that is partially predictive and could actually support assessments did not make it into a test guideline. And that also comes to the difficulties we have today in finding acceptance of mechanistic methods or small pieces in the puzzle that could actually help assessments without understanding the full solution. And to reach to a full solution, it takes a long time.”*

## Discussion

### Summary and discussion of results

This article considers the perspectives and experiences of people working in the regulatory toxicology system. Having representatives from multiple actors enables us to highlight system-level factors that could potentially be targeted through interventions. From a system perspective, one could conclude from the findings that NAMs will be used more effectively once more NAMs are being used in the regulatory toxicology system. However, advocating for more NAMs in the system is not the goal of this project. Rather, we are interested in the mechanisms ensuring an efficient transition towards using NAMs that offer actionable evidence and also in the mechanisms reducing the use of NAMs that do not create actionable evidence. Taken together, the observations from the three-day workshop and three online follow-up workshops highlight that in its current form, neither any of the technical aspects (infrastructure, processes, or technology), nor any of the social aspects (cultures, actors or goals) fully support the effective use of NAMs. More importantly and optimistically, our article highlights themes that should be reflected upon and potentially, could serve as basis for specific measures within the system. The insights gained based on the analysis of transcripts are rich and go beyond what could be summarised in this article. Nevertheless, we have extracted specific themes and preliminary implications for current practice, outlined in Table 2.

**Table 2:** Themes and preliminary implications

Theme	Preliminary implications
Current **incentive structures** • for regulators (e.g., avoidance of legal conflicts or conflicts of interest) • academia (e.g., acquiring funding and publishing papers) • and industry (e.g., getting market access through reliable regulatory acceptance of data sets) hinder the effective use of NAMs	Infrastructure, processes and cultures that incentivise the effective development, validation and use of NAMs for specific actors can act as promoters (e.g., funding and scientific recognition for validation-oriented research and development of regulatory relevant NAMs)
Different actors have different **goals** within the regulatory toxicology system. These differences in goals can act as inhibitors of the effective use of NAMs, as they determine incentive structures and promote siloing	This could somewhat be amended through dialogue and collaboration across siloes (e.g., events that aim at breaking up siloes), while considering infrastructure and processes that act as guardrails to mitigate conflicts of interests
Actors might have **expectations about the goals and incentive structures of other actors** (which may or may not be correct). This was described as another side-effect of siloing in the regulatory toxicology system and might have negative implications for the effective use of NAMs, as it might reduce trust in the system (e.g., misconceptions about other actors’ goals and a related lack of trust)	While trust among actors cannot be improved directly, the predictors of trust (e.g., perceived competence, value similarity, personal contact, alignment of goals) could potentially be tackled. Again, events that break up siloes can contribute to an improved understanding of other actors’ goals and incentive structures
System-wide and legal framework-specific likelihood and impact of **unwanted effects** of using NAMs (e.g. accepting positive results based on screening methods without follow-up testing) may act as inhibitors for the effective use of NAMs	Innovative initiatives that prevent, amend or mitigate these unwanted effects are needed
Clashes or at least **points of friction between scientific, regulatory, political, and legal infrastructure and processes** disrupt the effective use of NAMs	Potentially, placing targeted measures, such as sandboxes (e.g., forums of exchange for legal concerns), could resolve some of this friction
The effective development, validation and use of NAMs requires the development of **new skills for all actors,** which is hampered by the high level of siloing in the regulatory toxicology system	The speed of success through educational efforts could be supported by enhancing everyone’s understanding of the perspectives and pre-requisites of other actors in the regulatory toxicology system
In some instances, there seems to be an **imbalance between providing structure to reduce real and perceived uncertainty** (i.e., guidance, standard operating procedures, guidelines and operating manuals) and the **flexibility** required for the integration of continuously changing, new scientific insights and methodologies	Developing new formats for offering structure to regulators (e.g., tools to judge the validity of NAMs studies, assessment frameworks for modelling approaches)
**"First-mover hesitancy”** prevents different actors from utilising NAMs in an effective way	Coordinated global efforts and collaboration on regulatory decision-making frameworks and best-practice cases for NAMs could reduce this hesitancy

### Strengths and limitations of the methodological approaches

Qualitative data collection, analysis and interpretation used in this study have some inherent strengths (e.g., in-depth exploration of themes without the restrictions of closed-format questions) and challenges (e.g., inherent subjectivity in interpretation). Additionally, the selection of participants determines the quality of the collected data, and this is a potential limitation. We took care to ensure the participation of a heterogeneous and international group of people with recent or extensive experience working in the regulatory toxicology system. Moreover, the follow-up workshops aimed at compensating for the under-representation of certain groups in the workshop. The use of input presentations to spark discussions among participants has doubtlessly impacted the type of anecdotes that were shared with us. Jones et al. (n.d.) summarise some of the approaches that we used to ensure a lively exchange and open atmosphere. The analysis of such a vast data set also brings along some challenges, which were partly compensated by involving an interdisciplinary project group that met for weekly exchanges to contribute to and reflect upon the analysis work. Additionally, the progress was presented to and discussed with an advisory board monthly. A more in-depth discussion of the methodological strengths and weaknesses of our approach can be found in Jones et al. (n.d.).

## Conclusion

The perceived potential benefits of developing, validating, and using NAMs include “better protection of humans and the environment, the reduction of animal testing, and ultimately, a faster and more cost-effective test system for evaluating chemical safety” (Mathisen et al. 2024, p. 2299). The purpose of this article was to identify system-based promoters and inhibitors of the effective use of NAMs. The systems-thinking framework (Meadows 2008) focused the analysis and interpretation of the data in this article on the whole regulatory toxicology system as experienced by participants. The STS model (Davis et al. 2014; Hendrick 2006) allowed us to structure the themes that emerged from the qualitative analysis of this vast database. The next step will be to take these results into the next CHANGE workshop, the “Reflect Phase” in 2025. As its name suggests, the workshop will be an opportunity to reflect on the observations made about the different elements of the regulatory toxicology system and the most pressing themes outlined in this article. The present data set will be subjected to another data analysis that focuses more on causal loops, a construct from systems thinking that focuses on reinforcing and balancing effects, to reflect on system factors deemed important for the effective use of NAMs within the regulatory toxicology system that could be changed via targeted actions.

## Data Availability

For privacy protection of the workshop participants, pseudonymised transcripts are not made publicly available.

## Appendix

**Table 3:** Final code scheme used to code the workshop transcripts

Code/sub-code	Description	Anchor example
*Culture*	*Observations related to the culture of the system (not directly observable but experienced by members of the system)*	
Beliefs or assumptions	Observations about the beliefs and assumptions of the systems’ culture	(1) the entire academic system, of course, we’re really pushed to publish. ... It’s not really about the quality. It’s not really about the importance But several universities value much more the quantity of papers that you have (2) So, if something is not statutory, then I think the presumption always has been that the agency that is responsible can develop their own guidance but that it can have legal force, even if it’s not statutory (3) We see our [in] vivo test as a sort of golden standard, and some people do, but it’s rusty if it’s gold at all
Habits or behaviour	Observations about habits or behaviour within a particular culture of the system	(1) I think that if regulators use regularly academic data, and that was the case of the [chemical], especially because there were no regulatory data available. So, in that case, regulators need to fill the gaps with what they have. So, they use data produced by scientists (2) And the capacity to have sufficient information in materials and methods […] to replicate the study and to check if the results correspond to what is published is essential. And we are losing that in many publications. And there is no question of space because as a supplementary material, you can publish all the details. But it’s not the norm (3) The regulators in different organization, they don’t talk [to] each other. And we realize that the same chemical with different regulation
*Goals*	*Observations about the goals present in the system*	
Aims	Observations about the aims of the system or parts of the system (e.g., target, purpose, goal)	(1) We’re going to start out with our end goal. We're going to say, well, okay, the end goal is having a battery that is hopefully high throughput, or medium throughput at best. And we want to answer this question for different types of chemicals, different types of legislations, et cetera (2) For […] occupational health […], I think the ultimate goal is prevention maybe through prediction and protection
Incentives	Observations about incentives in the system or for parts of the system (e.g., values, valuable/relevant/important to xy)	(1) The funding I had from the [institution] many years ago forced some of us to bridge that gap by coming up with standard operating procedures even for statistical analyses, which typically that's not done with, really as a condition for funding And so, I think probably pushed us a bit toward making our published research A little bit more regulator-ready in terms of assessing what the findings were (2) Academics, of which I am one, are incentivized to explore new areas, to demonstrate novelty, and are rewarded in terms of advancement for being new and different
*Infrastructure*	*Observations about the infrastructure of the system e.g., what is put in place or present currently in the regulatory toxicology system* *Other than processes, infrastructure are structures in place to make the system work (e.g., buildings, roads, power supplies needed for the operation of a society. Infrastructure allows for processes to take place. They are not in constant flux, but more rigid than processes*	
Communication, dialogue or engagement	Observations about infrastructure put into place that promotes/hinders communication, dialogue and engagement among members of the system (e.g., knowledge exchanges, information provision, education)	(1) Given that they’re trying to write within word limits very often for a particular journal, and if you want to publish in one of the nature journals, they’re not going to give you an extensive amount of words to do it (2) Academics that are sort of putting their work out into the world and saying, yeah, it’s not really working trying to work with the regulators and so they put it forth in the media
Law, legal or regulatory aspects	Observations about legal or regulatory structures in the system (regulations, legal frameworks, laws)	(1) I don’t know if anybody else understands more about the law in [country] than I do, but from what I’ve understood there could be very big changes depending on [legal context], at least in [country] (2) And then we will put this in our law to say, okay, if this product is consumer product, then we will go to the different agency. If this industry chemical, then you go to like a [REACH]. And if this is other, so it just goes to different agencies, even they use the same chemical
Skills	Observations about the skills or infrastructure building up skills	(1) It’s not even an SOP, we heard from this morning, actually, now it’s [a template], so it’s even more elaborate. Well, it’s a shock. As an academic researcher, [I was] like, wow, how do I need to fill this out? It’s just because they’re not […] used to it (2) The managers they don’t follow the research trends they don’t follow the new methods and they just follow what is implemented by the regulators at the [geographical area] level for example and so it is a definite skill gap knowledge gap as well and yeah it’s a comfort gap
Funding	Observations about funding infrastructure, funding availability or institutions/structures that offer funding	(1) And I also related that the funding I had from the [institution] many years ago forced some of us to bridge that gap by coming up with standard operating procedures even for statistical analyses, which typically that’s not done with, really as a condition for funding
Supply chain	Observations about the supply chain	(1) There’s a quite new [NAM for a particular endpoint] and this cell line would be really nice to test it and we contacted them but we just got the response that it was not available yet so I don’t know so I guess that if a test guideline is adopted it must be available
*People*	*Observations about people in the system*	
Academia or the scientific community	Observations about academia or the scientific community, funding agencies or scientific journals or publishers	(1) Whatever the number of words you allow a scientist to put in a publication or outside in additional data, *a scientist* is producing something in a specific context for answering specific questions
Government	Observations about decision makers (e.g., risk managers, politicians)	(1) The decision makers are the policy and the heads, they make these decisions so include also the decision makers (2) Politicians want the vote from the general public, so they need each other
Industry	Observations about industry, manufacturers, producers, NPOs	(1) When I was working for industry, I used to work and interact with different stakeholders, regulators (2) And then on the other industry, they are losing market share, they are losing money
Legal	Observations about lawmakers, lawyers, judges	(1) […] I […] look at cases, especially the court cases, and it’s interesting then to look at what comes across as a language as a decision that it’s being made
Media or journalists	Observations about the media, journalists or other public commentators	(1) From a respectable journalist, everything was fine until you see that little thing a bit. And then, you know, so much you can do at that point (2) But I had a really nice discussion with this journalist on this. And in the end, he really got the point. And the piece that he published was pretty well balanced
Non-governmental organisations (NGOs)	Observations about NGOs (e.g., campaigning for public good)	(1) And NGO, at least in [country], there are so many NGOs, the small size, they try to feed themselves, they need money. And in order to accomplish their goal, they need size of issue, collaborate with politicians
Risk assessors	Observations about risk assessors, regulators, regulatory agencies	(1) The regulators in different organization, they don’t talk each other. And we realize that the same chemical with different regulation (2) Which is of course necessary for risk managers and assessors and there is a definite knowledge gap between what are [NAMs] how they are used for risk assessors and the managers
Public	Observations about the public in general, e.g., exposed communities, lay-people	(1) Politicians want the vote from the general public, so they need each other (2) Public engagement, because the goal also is public confidence, right?
*Processes*	*Observations about the technical & scientific processes of the system * *Other than infrastructure, processes can be in flux or change more readily*	
Regulatory	Observations about regulatory processes (e.g., testing requirements, reporting, reliability, validity, risk management)	(1) That challenging the substance of a decision, challenging the risk management decision relied upon on the evidence, is very difficult to do. It's very difficult to say that your exercise of judgment is erroneous. But what is much easier to do is to say that there are procedural flaws in the data, that your data is incomplete or insubstantial (2) When regulators take those data into their boxes or their constraints of regulation, ultimately you will have to translate somehow the work that has been done in a specific context to another context
Scientific	Observations about processes in science and research (e.g., problem formulation, NAM development, validation)	(1) There are lots of pressures, I think, on academic peer-reviewed writing, that make you push quite a lot of the detail of the study to the background (2) Funding mechanism […] is for innovation and if it's not just sustainable I see so many beautiful database, […] developed in the 5 years and then it was like ‘no maintenance’ so the government definitely needs to step in to facilitate this translation from the innovation
Social	Observations about social processes and interactions among individuals in the system	(1) I consider myself an academic. I’m not working in the regulatory domain, but it's sort of put such a divide and I know it’s not on purpose, right? But maybe we can think of a way how we can address these differences that there are. Let’s call them differences in a way that is a bit more, I don’t know, sort of without insulting, you know, each and every one of us
*Technical aspects*	*Observations related to the methodological and scientific technicalities in the system * *Other than infrastructure and processes, this is about the nitty-gritty details*	
Methodologies	Observations about specific tools/methods used in the technical or scientific system (e.g., sytematic reviews, meta-analysis, specific NAMs or combinations of NAMs, weight of evidence)	(1) Because of course, [institution] is only doing a hazard assessment, so there are no questions here of exposure at this point in time (2) People are still studying the same concept with meta-analysis and you can see why are we still studying this when the meta-analysis showed that’s for research where you can perform meta-analysis obviously
Tools	Codes about technical and scientific tools (e.g., Artificial Intelligence, Machine Learning, software, decision support tools, modelling)	(1) Have the AI do a check on all the published papers and where there is sufficient data on (2) And they develop a [tool] for trying to score the ingredients, the [chemical], and then to see how they can do a more eco-friendly with the formulation
*Solid gold*	*Use this code to call special attention to this observation later. Use it sparingly, only if you feel like this needs to be considered as a system-level factor or you have never heard this before* * Write a short memo and explain why you added this*	
*Unsure*	*This is the bin for everything that you are unsure about. If you feel like something is important but does not fit anywhere, put it in here*	

## Footnotes

1 The pseudonymised nature of the transcripts did not allow to identify individual speakers throughout the transcripts. Thus, the thematic issues summarised subsequently did not overly rely on frequency of mentions but rather on the content of the mentions. This ensured that observations that were repeated by the same person did not receive too much weight.
